# Environmental Sequencing Fills the Gap Between Parasitic Haplosporidians and Free‐living Giant Amoebae

**DOI:** 10.1111/jeu.12501

**Published:** 2018-02-02

**Authors:** Georgia M. Ward, Sigrid Neuhauser, René Groben, Stefan Ciaghi, Cédric Berney, Sarah Romac, David Bass

**Affiliations:** ^1^ Department of Life Sciences The Natural History Museum Cromwell Road London SW7 5BD United Kingdom; ^2^ Cefas Barrack Road, The Nothe Weymouth Dorset DT4 8UB United Kingdom; ^3^ College of Life and Environmental Sciences University of Exeter Stocker Road Exeter EX4 4QD United Kingdom; ^4^ Institute of Microbiology University of Innsbruck Technikerstraße Innsbruck 25 6020 Austria; ^5^ VÖR ‐ Marine Research Center at Breiðafjörður Norðurtangi Ólafsvík 355 Iceland; ^6^ Sorbonne Universités UPMC Université Paris 06 & CNRS UMR7144 Station Biologique de Roscoff Place Georges Teissier Roscoff 29680 France; ^7^ Present address: Matís ohf. Vínlandsleið 12 113 Reykjavík Iceland

**Keywords:** Ascetosporea, copepod, eDNA, Endomyxa, *Paradinium*, parasite

## Abstract

Class Ascetosporea (Rhizaria; Endomyxa) comprises many parasites of invertebrates. Within this group, recent group‐specific environmental DNA (eDNA) studies have contributed to the establishment of the new order Mikrocytida, a new phylogeny and characterization of Paramyxida, and illuminated the diversity and distribution of haplosporidians. Here, we use general and lineage‐specific PCR primers to investigate the phylogenetic “gap” between haplosporidians and their closest known free‐living relatives, the testate amoeba *Gromia* and reticulate amoeba *Filoreta*. Within this gap are *Paradinium* spp. parasites of copepods, which we show to be highly diverse and widely distributed in planktonic and benthic samples. We reveal a robustly supported radiation of parasites, ENDO‐3, comprised of *Paradinium* and three further clades (ENDO‐3a, ENDO‐3b and SPP). A further environmental group, ENDO‐2, perhaps comprising several clades, branches between this radiation and the free‐living amoebae. Early diverging haplosporidians were also amplified, often associated with bivalves or deep‐sea samples. The general primer approach amplified an overlapping set of novel lineages within ENDO‐3 and Haplosporida, whereas the group‐specific primer strategy, targeted to amplify from the earliest known divergent haplosporidians to *Gromia*, generated greater sequence diversity across part of this phylogenetic range.

THE retarian subphylum Endomyxa contains two major classes of parasites, which apparently evolved parasitism independently. Phytomyxea, which infect plant, algal, and stramenopile hosts (Neuhauser et al. 2014) are the sister taxon to predatory vampyrellid amoebae (Bass et al. 2009; Berney et al. 2013; Hess et al. 2012), while Ascetosporea, known members of which infect invertebrates, group in a clade also including large testate and reticulose free‐living amoebae, and several uncharacterized environmental lineages (Bass et al. 2009).

Ascetosporea comprises five orders—Haplosporida (Hartikainen et al. 2014a), Mikrocytida (Hartikainen et al. 2014b), Paramyxida (Ward et al. 2016), Claustrosporida and Paradinida (Bass et al. 2009). The first three orders include economically significant pathogens of molluscs and crustaceans, including the causative agents of oyster diseases MSX, QX, Aber disease and bonamiosis in oysters (*Haplosporidium nelsoni*,* Marteilia sydneyi*,* Marteilia refringens*, and *Bonamia* spp. respectively), as well as debilitating diseases of crabs (*Paramikrocytos canceri*) (Feist et al. 2009; Hartikainen et al. 2014a,b; Ward et al. 2016).

Little is known about Claustrosporida or Paradinida, and both orders were erected on the basis of very few characterized specimens. Only two genera have been proposed as paradinids, *Paradinium* and *Atelodinium*, both originally described by Chatton (1920) from the marine copepods *Acartia clausi* and *Paracalanus parvus*, although these paradinid genera were later synonymized (Chatton and Soyer 1973). The extensive study by Chatton (1920) focused on dinozoan (“péridinien”) parasites generally, but included genera Chatton considered related to but not necessarily belonging to the Dinozoa: Paradinidae (*Paradinium* and *Atelodinium*), Ellobiopsidae (*Ellobiopsis*,* Staphylocystis*,* Ellobiocystis* and *Parallobiopsis*) and Blastuloidae (*Neresheimeria* (=*Lohmanella*)). It is now known that *Ellobiopsis* groups phylogenetically within Dinozoa (Gomez et al. 2009), and *Amoebophyra*, which was affiliated with *Neresheimeria* in Blastuloidae by Neresheimer (1904), is a syndinian. Both of these are therefore dinozoan. Chatton (1920) notes characteristics of paradinids that could not only indicate a relationship with syndinians but also identifies many differences between them.

The first sequence data for *Paradinium* was published by Skovgaard and Daugbjerg (2008), showing moderate support for a sister relationship with haplosporidia. 18S rDNA sequences were generated for two *Paradinium* lineages: *P. poucheti* from *Oithona similis* (PaOi01) and an undescribed *Paradinium* sp. from *Euterpina acutifrons* (PaEu41) (i.e., two different copepod host species). Two sequences from parasites of the spot prawn *Pandalus platyceros* (Bower and Meyer 2002) formed a weakly supported clade with *Paradinium* in the Bayesian phylogeny of Skovgaard and Daugbjerg (2008). Bower and Meyer (2002) reported that the spot prawn parasite (SPP) was phylogenetically related to haplosporidians, which is confirmed by Reece et al. (2004).

Resolving the phylogenetic position of parasitic lineages is often complicated by long branches on trees caused by divergent sequences, compounded by low levels of taxon sampling in groups that are difficult to sample. The lineage sampling of Endomyxa was increased by using group‐specific 18S primers in Bass et al. (2009), revealing novel environmental clades (ENDO‐2 and ‐3) clustering in a moderately supported clade with Haplosporida and the giant testate marine amoeba *Gromia* and reticulate amoeba *Filoreta*. Further analyses indicated that *Paradinium* and SPP grouped with environmental clade ENDO‐3 (Bass et al. 2009).

Subsequent studies investigating the diversity of Ascetosporea demonstrated that the use of PCR primers designed specifically to divergent groups reveal further novel diversity, for example, of haplosporidians and mikrocytids (Hartikainen et al. 2014a,b) and paramyxids (Ward et al. 2016). These studies also showed that extracting DNA directly from putative hosts of these parasites is a good way of accessing additional diversity, and suggesting host–parasite associations. As Endo‐2/3, *Paradinium* and SPP occupy interesting evolutionary positions between free‐living and parasitic lineages, and likely also harbour unknown diversity, we designed primers to amplify from basal haploporidians (specifically the haplosporidian parasite of *Ruditapes decussatus* AY435093) to Endo‐2 sequences DQ504354/EU567273. We refer to this phylogenetic range as “paradinids and earlier diverging Ascetosporea” (PEDA).

Copepods are the most abundant metazoans in the marine plankton, and indeed on earth, underpinning the marine trophic network (Turner 2004). Their role as reservoirs and vectors of parasites of larger invertebrates is increasingly recognized (e.g. Arzul et al. 2014), and a longer standing interest in their symbionts has resulted in a body of work which suggests that their protistan parasites are dominated by dinozoans (Skovgaard and Saiz 2006; Skovgaard et al. 2005, 2007, 2012), and that they are also basibionts for many suctorian ciliates (Gregori et al. 2016). Nonetheless *Paradinium* species have been observed parasitizing a number of copepod species, and studies of seasonal occurrence (Chatton and Soyer 1973; Skovgaard and Saiz 2006) suggest the parasites may have a high prevalence (up to 35%). Although we cannot say that all lineages related to *Paradinium* are also parasites of copepods, we propose that ascetosporean parasites of crustaceans are much more diverse in terms of lineage richness and ecology than previously recognized.

## Materials and Methods

To investigate diversity of PEDA, we used complementary sequence generation methods using four primer strategies on a broad range of sample types: basal ascetosporean‐targeted (PEDA) PCR primers amplicons generated from global water and sediment samples and invertebrate tissues, endomyxan‐biased primers applied to European coastal marine water and sediment samples, and two sets of broadly targeted eukaryote‐wide primers applied to bivalve and associated water column samples from Iceland. The PEDA amplicons were cloned and Sanger sequenced in order to provide longer sequence reads for phylogenetic analyses. Different regions of the 18S rRNA gene were targeted: the eukaryote‐wide and endomyxan‐biased primers amplified the V4 region (recognized as generally the most variable 18S region suitable for phylogenetic interpretation; Stoeck et al. 2010), the V5–9 regions were amplified by the targeted PEDA primers (determined by availability of sites for primer design and derived by modifying the comparable haplosporidian primers used by Hartikainen et al. 2014a). An additional eukaryote‐wide amplicon (V3) was used in parallel with the eukaryote‐wide V4 primers to test their utility for detecting parasites associated with potential hosts.

### Sample collection and nucleic acid extraction

#### Environmental samples

Water and sediment samples were collected from sites in Weymouth, UK (Fleet Lagoon, < 10–30 ppt salinity; 50°35′N, 2°28′W, and Newton's Cove; 50°34′N, 2°22′W) in June and October 2011 and April 2012, and three sites along the estuary of the River Tamar, UK (Cremyll Ferry; 50.35°N 4.17°W, Wilcove, 50.387°N 4.201°W; and Neal Point 50.443°N 4.204°W) in June 2013, as described in Hartikainen et al. (2014a,b) and Ward et al. (2016). Water samples were similarly collected (omitting the 0.45‐μm filtering step) from sites in Sabah, Borneo, Malaysia in December 2011, the Western Cape, South Africa, and Florida, USA in June 2014 as described in Ward et al. (2016).

Sediment and water samples were collected from coastal locations near Blanes, Spain (Balearic Sea), Oslo, Norway (Skagerrak, Oslofjorden), Naples, Italy (Tyrrhenian Sea) Varna, Bulgaria (Black Sea) as part of the BioMarKs Consortium (Logares et al. 2014; Massana et al. 2015). The water was then sequentially filtered and DNA and cDNA generated as in Massana et al. (2015). The deep‐sea water samples were described in Bass et al. (2007).

#### Invertebrate tissue samples

Tissue from abundant invertebrates, including amphipods, mussels, nudibranchs, polychaetes and crabs, was collected from the sites in Weymouth, the Tamar estuary and Florida, and preserved in 100% ethanol, as described in Hartikainen et al. (2014b) and Ward et al. (2016). DNA was extracted from the tissue samples using the DNeasy Blood & Tissue Kit (Qiagen).

Blue mussels (*Mytilus edulis*) and Icelandic scallops (*Chlamys islandica*) were collected together with corresponding sea water samples near the islands of Kiðey and Purkey in Breiðafjörður, West Iceland, in June and August 2010, July and August 2011, and January 2012. Guts were dissected out of the bivalves and their contents collected in 100% ethanol until further processing. DNA from bivalve gut contents and corresponding water samples was isolated using the PowerSoil DNA Isolation Kit (MoBio Laboratories).

### PCR, sequencing and sequence processing

#### 18S rDNA V5–V9 region amplicons

Primers were designed to amplify the V5–V9 region of the SSU gene based on all known sequence data from basal ascetosporean lineages, as of June 2013. The primers were designed to detect diversity between ENDO‐2 (DQ504354/EU567273) and the deep‐branching haplosporidian parasite of *Ruditapes decussatus* (AY435093), inclusive of known environmental sequences and crustacean parasites but excluding *Gromia*,* Filoreta* and most Haplosporida. These primers were applied to water and sediment samples from around the world, and invertebrate tissue from the U.K. and Florida.

A nested PCR protocol was designed, using primers V4fAsce and SB1n for the first round, followed by V5fAsce and EndoR1 for the final round (Table 1). All PCR reactions were conducted in 20 μl final volumes with 1 μl of template DNA and final concentration of 0.5 μM of each primer, 0.4 mM dNTPs, 2.5 mM of MgCl_2_, 1× Promega Green Buffer and 0.5 U of Promega GoTaq. All PCR reactions were carried out in an ABI Veriti Thermal Cycler. Cycling conditions for both rounds of the nested protocol consisted of denaturation at 95 °C for 5 min, followed by 30 cycles of 95 °C denaturation for 30 s, annealing at 65 °C for 1 min and an extension step at 72 °C for 1 min, followed by a 10 min final extension at 72 °C, then stored at 4 °C. Amplicons from environmental samples were pooled by sample type and site and purified using polyethylene glycol and ethanol precipitation. Clone libraries were prepared using the StrataGene cloning kit (Agilent Technologies, Santa Clara, CA, USA).

**Table 1:** Sequences of primers used to generate amplicons covering different regions of the 18S rRNA gene. “Pool ratio” column indicates ratio of reverse primers added to V4 Endomyxan‐biased reverse primer pool (final working stock concentration 10 μM)

Gene region	Target taxa	Samples screened	Primer name	Primer orientation	Primer sequence (5′–3′)	Pool	Pool ratio	References
V3	General Eukaryote	Icelandic bivalve guts and water	MED454f	Forward	ATT AGG GTT CGA ATT CCG GAG AGG			Medinger et al. (2010)
			MED454r	Reverse	CTG GAA TTA CCG CGG STG CTG			
V4	General Eukaryote	Icelandic bivalve guts and filtered water	3NDf	Forward	GGC AAG TCT GGT GCC AG			Bråte et al. (2010)
			V4EukR2	Reverse	ACG GTA ATC TRA TCR TCT TCG			
V4	Endomyxa‐biased Eukaryote	European coastal filtered water and sediment DNA and cDNA	S6f	Forward	GAG GRM AAG YCT GGT GCC AGC ASC			
			EndoR0	Reverse	CGA CTT CTC CTT CCT CTA AAT GAT AAG	EndoR mix	1	
			EndoR1		CGA CTT CTC CTT CCT CTA ARY RDT AWG		1	
			EndoR2		CGA CTT CTC CTT CCT CTA ARY GHY WWG		1	
			EndoR3		CGA CTT YTC CTT CCT CTA RAT RDY AWG		1	
			V4fEuk	Forward	CCA GCA SCC GCG GTA AYW CC	V4f Mix	1	
			V4fEnd		GTG CCA GCA GCC GCG GTA AYA		1	
			C0	Reverse	CAC CAC CCA TAG AAT CAA GAA AGA TCT TCA	S1256r Mix	16	
			48		CAC TAH CCA TAG AAT CAA GAA AGR KCT KCA		4	
			Va		CAC YAY CCA TAG AAT CAA GAA AGA TCK TCA		2	
			Ph		CAC YAC CCA TAG AAT CAA GAA AGA GCT KCA		2	
			Ha		CAC YAT KCA TAG AAT CAW GAA AGA ACT TBA		2	
			Fi		CAC CAC CCA YAG AAT CAA GAA AGR TCT TCA		2	
			Pl		CAC CAC CGA AGT GAT CAA GAA AGA KCT KCA		1	
			12		CAC CAM CCA WAG AAT CAA GAA AGA TCT GCA		1	
			Re		CAC CAM CCA TMR AAT CAA GAA AGA TCT TCA		1	
			Gr		CAC CAC CCA TAW AAT CAA GWA AGA KCT KCA		1	
V5–V9	Paradinids and earlier diverging Ascetosporea (see grey shaded area on Fig. 1)	Coastal and littoral water and sediment, DNA and cDNA; invertebrate tissue and incubation samples	V4fAsce	Forward	GGA ATA ATA WGA TAG GAC TTC RGC A			
			Sb1n	Reverse	GAT CCH TCY GGA GGT TCA CCT ACG			
			V5fAsce	Forward	GYT CRG CAC CKT ATT YGA GAA ATC A			
			EndoR1	Reverse	CGA CTT CTC CTT CCT CTA ARY RDT AWG			

#### 18S rDNA V4 region amplicons

Two different sets of primers amplifying the V4 hypervariable region of the SSU gene were applied to different sample sets. The Icelandic bivalve gut tissue and water samples were amplified with the general eukaryote 3NDF and V4eukR1 primers as described in Bråte et al. (2010). In addition to the taxon‐specific sequences, the primers also contained directional GS FLX Titanium primer and key sequences and, in case of the forward primer, 14 different Multiplex Identifier (MID) sequences to allow barcoding and multiplexing of samples. PCR reactions were done in triplicate, pooled, cleaned using AMPure magnetic beads (Agencourt) and quantified using the Quant‐iT PicoGreen ssDNA Assay Kit (Thermo Fisher Scientific) before being pooled in equimolar amounts according to their MIDs for emulsion PCR and pyrosequencing using the GS FLX Titanium chemistry. A whole PicoTiter plate was used for the analysis, separated into eight regions with 14 different samples per primer pair and four different primer pairs used in each region. All methods were used according to the manufacturers’ instructions.

Endomyxa‐biased V4 amplicons were generated from European sediment and water samples using a cocktail of primers in a nested PCR protocol: first round—forward primer s6f and reverse pool EndoRmix; second round—forward pool V4fmix and reverse pool s1256Rmix for the nested round (Table 1). Reaction mixtures were of the same composition as used for the V5–V9 PCRs. Cycling conditions: first round—initial denaturation at 95 °C for 3 min, followed by 36 cycles of 95 °C denaturation for 30 s, 66 °C annealing for 30 s and a 72 °C extension step for 1 min 30 s. Final extension at 72 °C for 10 min before storage at 6 °C. Second round: these conditions were altered to increase the number of cycles to 39, and the annealing temperature was increased to 67.5 °C. Expected amplicon size was 700–900 bp. The forward primers comprised the relevant sequences in Table 1, the Roche 454 A adaptor, and either one of three three‐nucleotide MIDs or no MID. These four bioinformatically sortable conditions were distributed across three half‐runs to enable 16 separate libraries to be sequenced: DNA/cDNA, water column/sediment, in all combinations each for four sampling sites (a, b, c, d).

#### 18S rDNA V3 region amplicons

Amplification of the V3 regions of the SSU gene from Icelandic bivalve gut and water samples were carried out as given in Medinger et al. (2010). The unnamed primers in that publication were designated the names Med454f and Med454r for the forward and reverse primer respectively.

#### Sequence processing and definition of OTUs

Icelandic bivalve gut tissue and water samples: the 454 amplicons were processed following the 454 Standard Operating Procedure (SOP) for mothur (http://www.mothur.org/wiki/454_SOP; accessed September 2012) using mothur Version 1.27.0 (Schloss et al. 2009, 2011). Quality control parameters were chosen according to the 454 SOP with a minimum amplicon length of 100 bp and using chimera.uchime for chimera detection. Alignment of the amplicons in mothur was done using the SILVA‐compatible reference alignment for eukaryotes (http://www.mothur.org/wiki/Silva_reference_files) based on SILVA v102 (Pruesse et al. 2007; Quast et al. 2013). Taxonomic identification of amplicons used the classify.seqs command with default settings on a mothur‐compatible dataset of 71787 eukaryotic sequences derived from SILVA release “SSURef 111” as reference (file available on demand from the authors). All sequences identified as belonging to the Ascetosporea were extracted from the whole dataset for further phylogenetic analyses.

Endomyxa‐biased V4 amplicons: the raw sequence files (SFF files) were processed using QIIME v 1.8.0 (Caporaso et al. 2010). The demultiplexing and quality filtering steps were done using default parameters except for minimum read length (150 bp instead of 200 bp) and maximum primer mismatches (three instead of zero) to allow for wobbles and ambiguous bases in the primers used (Table 1). Sequences were trimmed to 100 bp, then dereplicated and singletons were removed. OTU clustering of the remaining sequences was done with a threshold of 97% sequence similarity using USEARCH version 9 (Edgar 2013). Finally, taxonomy was assigned using the BLAST algorithm (Altschul et al. 1990) against the PR2 reference database (release 191, Guillou et al. 2013) and an OTU table was created. Based on this OTU table the untrimmed representative sequences for all ascetosporean OTUs have been extracted from the remaining dataset after the quality filtering steps. These “full‐length” sequences were used for subsequent analyses.

### Phylogenetic analyses

Three 18S alignments were produced (V3, V4 and V5–9) using the sequences generated as above aligned with all available basal ascetosporean, haplosporidian, gromiid and reticulosid and closely related environmental 18S sequences from GenBank, identified by blastn searches in January 2016. In each case sequences were aligned using the e‐ins‐I algorithm on the MAFFT server (Katoh and Standley 2013), terminal gaps were trimmed, the alignment was refined manually, and regions of ambiguous alignment and large indels were removed (masked) by eye. Bootstrapped Maximum Likelihood (ML) trees were then calculated via the Cipres Science Gateway server (Miller et al. 2010) using RAxML BlackBox version 8.2.9 (Stamatakis 2014; Stamatakis et al. 2008) (GTR + CAT; all parameters estimated from the data); bootstrap values were mapped onto the highest likelihood tree obtained. Closely related sequences were then further collapsed into molecularly defined (OTUs) using the criterion that > 3 nucleotide differences (including gaps) in any single variable region in the amplicon defined a unique OTU, as used by Hartikainen et al. (2014a) for the analysis of haplosporidian environmental sequence data.

The ML trees were then re‐run, and corresponding Bayesian consensus trees were constructed using MrBayes v 3.2.5 (Ronquist et al. 2012). Two separate MC^3^ runs with randomly generated starting trees were carried out for 2M generations each with one cold and three heated chains. The evolutionary model applied included a GTR substitution matrix, a four‐category autocorrelated gamma correction and the covarion model. All parameters were estimated from the data. Trees were sampled every 1,000 generations. 500,000 generations were discarded as “burn‐in” (trees sampled before the likelihood plots reached a plateau) and a consensus tree was constructed from the returning sample. ML bootstrap values were plotted onto the Bayesian topology on Fig. 3. The sequences are deposited in Genbank (accession numbers: MG746635‐778).

## Results

Analysis of data generated using general eukaryote primers targeting the V3 regions of the SSU gene resulted in 229 of 170,169 (0.13%) sequences belonging to Ascetosporea. Analysis of data generated from the same samples using V4‐targeted primers produced 101 of 62,914 (0.16%) ascetosporean sequences. Between 1 and 6% of sequences generated using Endomyxa‐biased V4 primers belonged to Ascetosporea. The group‐specific PEDA primers, targeting the V5–V9 regions of the SSU gene, produced only ascetosporean sequence types.

**Figure 1:**
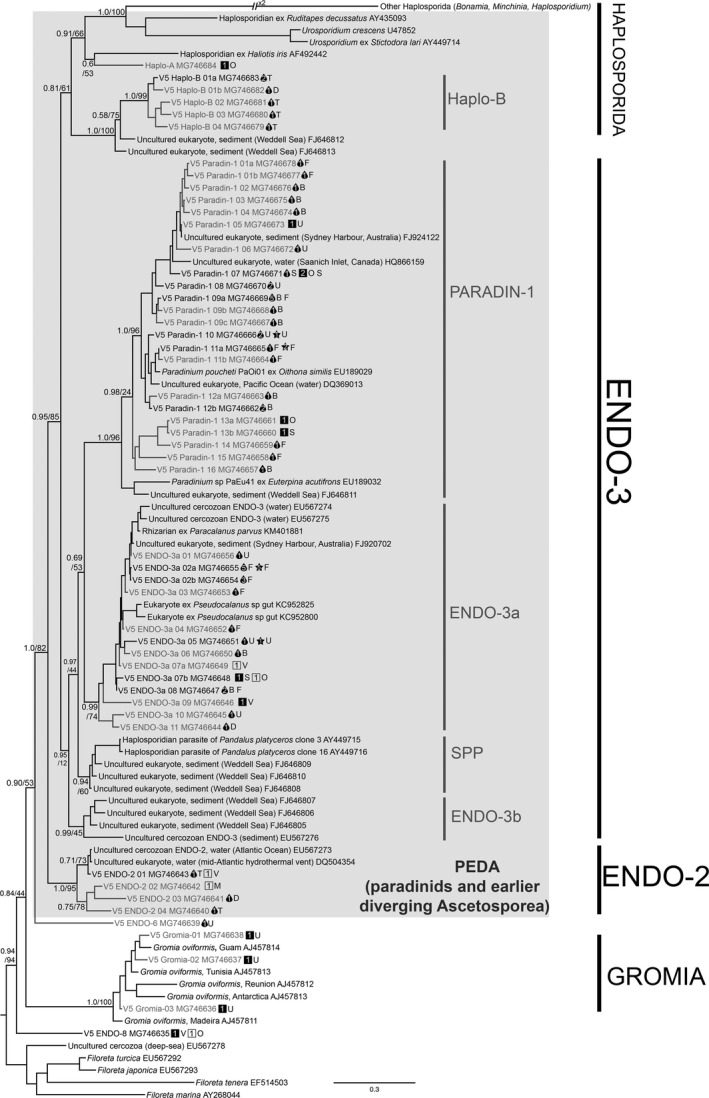
Bayesian phylogenetic analysis of 18S rDNA V5–V9 region amplicons generated in this study in the context of all available related GenBank sequences, plus representative haplosporidians, Gromia and Filoreta. The full length of GenBank sequences were used for the analyses. Values on nodes represent Bayesian Posterior Probabilities and Maximum Likelihood boostrap values respectively. Numbers in symbols to the right of sequence name show the number of libraries in which each OTU was detected. Squares = sediment (filled = DNA template, open = cDNA template), drop‐shaped = filtered water, stars = invertebrate tissue and incubation samples. Letters to the right of these indicate (marine) sampling sites: B = Borneo, D = Weddell Sea (deep), F = Florida, USA M = Mediterranean Sea off Barcelona, Spain, O = Oslofjord, Norway, S = South Africa, T = filtered water from near Titanic wreck, U = U.K. (Newton's Cove and Fleet Lagoon, Dorset; Tamar estuary, Devon), V = Black Sea off Varna, Bulgaria. “ENDO‐x” labels of lineages/clades derived from Bass et al. (2009). The shaded area labelled “PEDA” shows the target region of the V5–9 primer set devised for this study (Table 1).

**Figure 2:**
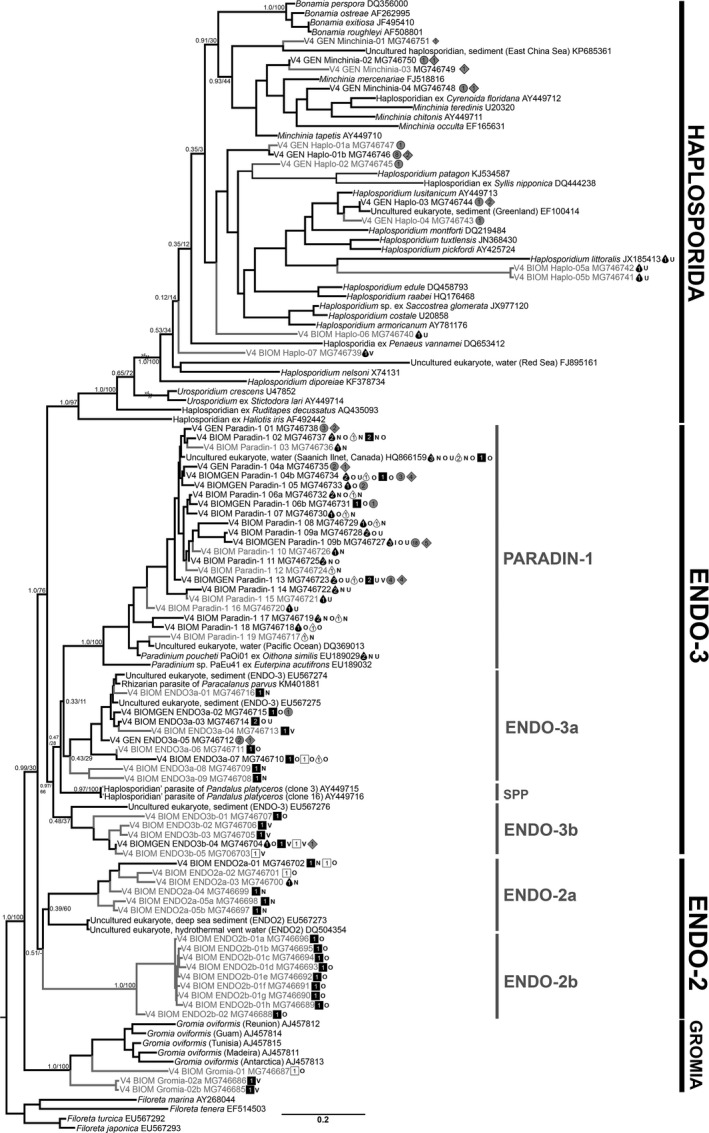
Bayesian phylogenetic analysis of 18S rDNA V4 region amplicons generated in this study by endomyxan‐biased primers and by broadly targeted V4 region primers. All available related GenBank sequences are also included, plus representative haplosporidians, *Gromia*, and *Filoreta*. The full length of GenBank sequences were used for the analyses. Values on nodes represent Bayesian Posterior Probabilities and Maximum Likelihood boostrap values respectively. Numbers in symbols to the right of sequence name show the number of libraries in which each OTU was detected. Squares = sediment (filled = DNA template, open = RNA template), drop‐shaped = filtered water, circles = scallop gut tissue, diamonds = mussel gut tissue. Letters to the right of these indicate (marine) sampling sites: B = Borneo, D = Weddell Sea (deep), F = Florida, M = Barcelona, O = Oslofjord, Norway, S = South Africa, T = filtered water from near Titanic wreck, U = U.K. (Newton's Cove and Fleet Lagoon, Dorset), V = Black Sea off Varna, Bulgaria (see Table 1 for site details). “ENDO‐x” labels of lineages/clades derived from Bass et al. (2009).

**Figure 3:**
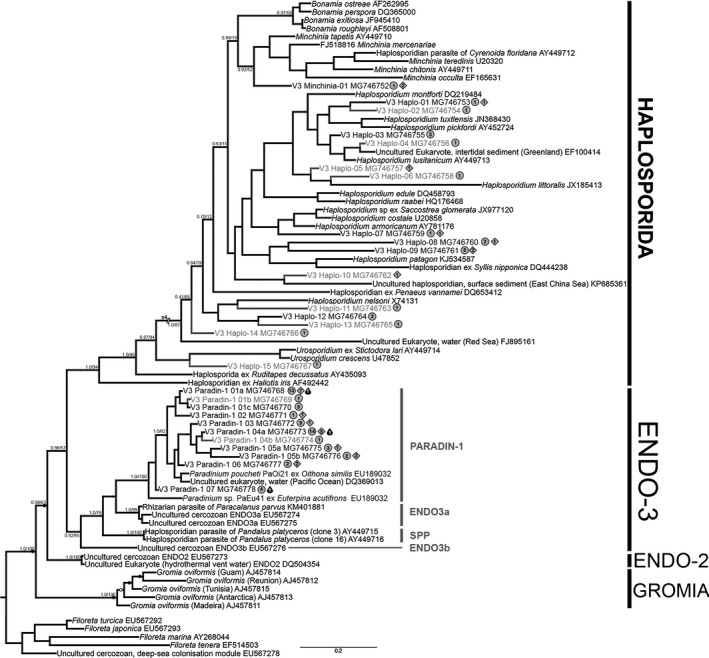
Bayesian phylogenetic analysis of 18S rDNA V3 region amplicons generated in this study from Icelandic samples in the context of all available related GenBank sequences, plus representative haplosporidians, Gromia and Filoreta. The full length of GenBank sequences were used for the analyses. Values on nodes represent Bayesian Posterior Probabilities and Maximum Likelihood boostrap values respectively. Numbers in symbols to the right of sequence name show the number of libraries in which each OTU was detected. Drop‐shaped = filtered water collected at Icelandic bivalve beds, circles = scallop gut tissue, diamonds = mussel gut tissue. “ENDO‐x” labels of lineages/clades derived from Bass et al. (2009).

Separate phylogenetic analyses of the V5–V9, V4 and V3 alignments produced three trees (Figs 1, 2, 3 respectively). The V5–V9 tree includes OTUs generated by the PEDA primer set from global littoral water, sediment and invertebrate tissue samples, and European coastal sediments (lineages labelled “V5” on Fig. 1). The PEDA phylogenetic range is also shown on Fig. 1. The V4 analysis, shown in Fig. 2, combined data from two primer sets: lineages labelled V4 BIOM, amplified from European coastal sediments and water samples (endomyxan‐biased primers) and lineages labelled V4 GEN (Icelandic mussel and scallop gut tissue and associated water samples; general eukaryote primers). Lineages labelled V4 BIOMGEN were amplified by both primer sets. The V3 tree includes operational taxonomic units (OTUs) generated from Icelandic mussel and scallop gut tissue and water samples using the V3 general eukaryote primers (Fig. 3). On all three trees, OTUs detected in a single library are shown in grey.

The sequences amplified by the different primer/sample strategies grouped into seven clades, labelled (where present) on Figs 1, 2, 3 as PARADIN‐1, ENDO‐3a and b, SPP (together forming ENDO‐3), ENDO‐2a and b, and HAPLO‐B. ENDO‐3 was sister to Haplosporida in all analyses with moderate support, ENDO‐2 being sister to both of them (also moderate support). HAPLO‐B is basal to all known haplosporidians.

Other novel sequence types were generated outside of ENDO‐3 and ENDO‐2: (1) the V5–V9 primers (Fig. 1) amplified a divergent lineage ENDO‐6 from a single littoral water sample from the U.K., grouping between PEDA and the closest known relative, *Gromia*. (2) ENDO‐8, detected independently from marine sediments from Oslo (cDNA) and Varna (DNA), grouping between *Gromia* and *Filoreta*. (3) three lineages from the Fleet lagoon sediment grouping within the Gromia radiation in the V5–V9 analysis (Fig. 1), and three further novel sequence types grouping as sisters to *Gromia* (V4 BIOM Gromia‐01 and ‐2), also from Oslo and Varna sediments, in the V4 analysis (Fig. 2).

### Diversity within ENDO‐3

#### PARADIN‐1

Sequences belonging to PARADIN‐1 were amplified from many sites (Florida, Borneo, U.K., Italy, Norway, South Africa) and sample types by all four primer sets (Figs 1, 2, 3). It includes *Paradinium poucheti* (PaOi21) and *Paradinium* sp. (PaEu41) from Skovgaard and Daugbjerg (2008). These are separated by a fundamental bipartition in the clade, with all of the environmental diversity detected in this study belonging to the clade including PaOi21—we detected no other members of the clade including PaEu41. A sediment‐derived sequence from the Weddell Sea (FJ646811) groups as sister to this sequence in Fig. 1 (the sequence was omitted from phylogenetic analyses covering the V3 and V4 analyses as these regions are not covered by the sequence). In the *P. poucheti* subclade the majority of lineages detected came from water column DNA samples—none came from BioMarKs coastal sediment cDNA samples. Two lineages closely related to *P. poucheti* (V5 Paradin‐1 10 and V5 Paradin‐1 11a) were also detected in ascidian tissue (Fleet lagoon) and marine gastropod and oyster incubations (Florida), respectively, as indicated by star annotations on Fig. 1. No other sequences in PARADIN‐1 derived from invertebrate (‐associated) samples in the V5–V9 analysis, however, PARADIN‐1 sequence types were commonly amplified from scallop and mussel tissue samples using general eukaryote V4 primers (Fig. 2).

#### ENDO‐3a,b and SPP clades

ENDO‐3 was originally defined in Bass et al. (2009) on the basis of three environmental sequences: EU567274‐6. Neither the spot prawn parasite (SPP) nor any confirmed *Paradinium* sequence was included in that paper, therefore, the integrity of the (moderately well supported) ENDO‐3 was not further tested. All of our current trees show that the SPP sequences makes ENDO‐3, as originally described, paraphyletic, so we have re‐named lineages related to the three above as ENDO‐3a (EU567274/5) and b (EU567276). Both ENDO‐3a and SPP contain uncharacterized parasites of crustaceans (the copepod *P. parvus* and prawn *P. platyceros* respectively), whereas the lifestyle of ENDO‐3b remains unknown. In the V5–V9 analysis (Fig. 1), ENDO‐3a also contains previously detected sequences from the gut of *Pseudocalanus* spp. copepods (KC952800 and KC952825). We detected novel ENDO‐3a lineages mostly not only from water column DNA but also sediment DNA and cDNA. V5 ENDO‐3a 02a was detected in crab, sea urchin and zooplankton incubations, all from Florida, and V5 ENDO‐03a 05 from an edible mussel incubation (Tamar, UK). Lineages in this clade were detected world‐wide, from Florida, U.K., the Black Sea, Norway and in the Drake Passage. In the V4 analysis (Fig. 2), sequences grouping within this clade were mostly not only from European coastal sediments but also from mussel and scallop gut samples.

No sequences generated by any primer set grouped with SPP in any analysis, and ENDO‐3b was only detected by the two primer sets used for the V4 analysis. However, the V5–9 tree (Fig. 1) is informative as it shows that both SPP relatives and ENDO‐3b are present in deep (c. 4,900 m) Weddell Sea sediments (Lecroq et al. 2009) (all the original ENDO‐3 sequences in Bass et al. (2009) were from a range of deep‐sea samples).

### ENDO‐2

ENDO‐2 was detected by both V5–9 and V4 primer sets, from water and sediment samples, DNA and cDNA, but not from any host‐associated samples. No sequences within this clade were amplified using the V3 primer set. Although this lineage has been annotated as haplosporidian (DQ504354) on GenBank, all of our phylogenetic analyses show that it is not, and in fact forms a separate clade branching between ENDO‐3 and *Gromia*, and so this mislabelling has been omitted from all figures. As is the case for ENDO‐3b, there is no morphological evidence for this clade.

ENDO‐2 V4 amplicons cluster in three robust but weakly mutually related clades, two of which were unknown prior to this study (ENDO2a and ENDO2b). All sequence types within ENDO‐2b were from the same library (Oslo sediment DNA), however, following completion of phylogenetic analyses further BLAST searches of these sequence types against the NCBI GenBank database recovered two environmental sequence types, from Adventfjorden in Norway, showing high sequence identity (98–99%) to V4 BIOM ENDO2b‐01a (KT812216) and V4 BIOM ENDO2b‐02 (KT810733).

Although true Haplosporida are not the focus of this work it is worth noting that all three primer sets detected diversity in this clade. The broadly targeted V3 and V4 primers amplified a wide range of haplosporidians, which cannot be directly compared to those in Hartikainen et al. (2014a) as the amplicons do not overlap. The V5–9 primer region does overlap but was not targeted to haplosporidians. However, an interesting novel clade, Haplo‐B, sister to all other Haplosporida, was amplified from deep‐sea samples only (from near the wreck of the Titanic), and groups on Fig. 1 with other deep‐sea samples (c. 4,900 m) sequenced as part of a study of komoiacean foraminifera in the Weddell Sea (Lecroq et al. 2009).

## Discussion

This study is further evidence that PCR primers targeted to defined phylogenetic ranges provide a powerful tool for revealing diversity that more broadly targeted primers either fail to amplify or only produce as a small proportion of large sequence datasets. Here, we designed a primer strategy to investigate the Paradinida, the ascetosporean order for which only a small amount of sequence data exist, and also to populate the region of the ascetosporean phylogeny between the free‐living amoebae *Gromia* and *Filoreta* and basal haplosporidians.

We reveal a major novel endomyxan clade, ENDO‐3, robustly sister to Haplosporida. Morphological information is available for only two subclades of ENDO‐3: two lineages whose morphology is entirely concordant with *Paradinium* (PaEu41 and PaOi01) within PARADIN‐1, and the SPP. *Paradinium* has a filo‐plasmodial trophic stage which develops into a gonosphere (plasmodial cell mass), from which flagellated dispersal stages are formed. Such plasmodial types and free‐swimming flagellated zoospores are so far unknown in haplosporids. Other lineages within PARADIN‐1 have also been detected in planktonic environmental samples and therefore may represent a large radiation of copepod parasites that includes ENDO‐3a, although the strongest evidence so far for the latter is their strong planktonic bias and detection in the gut of *Pseudocalanus* spp., and the inclusion within this clade of an uncharacterized parasite of the copepod *P. parvus*.

Earlier diverging clades within ENDO‐3 include SPP, which is the only other lineage between haplosporids and *Gromia* and *Filoreta* for which morphology is known. Similarly to *Paradinium*, SPP does not possess haplosporosomes or lidded spores (as do haplosporids), but SPP differs from *Paradinium* in having unicellular, nonflagellated sessile trophonts developing from undivided plasmodia. Loss of the flagellate condition seems to be common in Endomyxa: the testate amoeba *Gromia* has flagellated gametes but its closest relative, the naked reticulate amoeba Filoreta apparently does not. In all of our trees the earliest diverging lineage in ENDO‐3 was ENDO‐3b, known only from marine benthic samples, some from great depth.

The sister clade to ENDO‐3 plus Haplosporida in all analyses is ENDO‐2, again only known from benthic or near‐benthic habitats, including low oxygen (Varna) and deep‐sea samples. Although evolutionary relationships strongly suggest that ENDO‐3a is parasitic/symbiotic, and that ENDO‐3b might be, the intermediate branching position of ENDO‐2 between the free‐living amoebae and ENDO‐3 provides less basis for such a hypothesis. The V4 dataset (also the largest in terms of sequence number and sample coverage) also contained ENDO‐2b, so far only detected in sediments from Oslo, and the V5–V9 dataset contains ENDO‐6, whose phylogenetic position within the Ascetosporea plus Gromia clade is unresolved (Fig. 1). ENDO‐8 may be the closest relative to *Gromia* and *Filoreta* revealed by the study; therefore, we suggest it may resemble those or is a novel amoeboid form. Environmental OTU association analyses (e.g. interactome, Science; Lima‐Mendez et al. 2015) may suggest potential hosts for ENDO‐2 if it is parasitic, but direct evidence is required to prove such an association, for example, via a histological‐molecular survey of invertebrates from habitats in which ENDO‐2 is known or likely to occur.

The novel deep‐branching haploporids detected (Figs 2 and 3) expand the known ecological range of this order. Many of these were derived from the Icelandic bivalve‐associated samples and may represent previously unknown parasites of those bivalves (e.g. V3 Haplo‐11, ‐12 and ‐13, related to *H. nelsoni*; Fig. 3). V3 Haplo‐15 (Fig. 3) might be a hyperparasite, like its relative *Urosporidium*. The even deeper, exclusively branching, deep‐water clade HAPLO‐B (Fig. 1) may represent a radiation of parasites of an unknown (or at least unsampled) bathyphilic invertebrate. Additionally/alternatively some of the Weddell Sea sequences, which were sampled in association with the foraminiferans *Normanina conferta* and *Septuma ocotillo*, may be symbionts of those much larger, related protists, in a similar system to the high protistan diversity recently revealed to be associated with radiolarians (sister to foraminifera within the phylum Retaria) (Bråte et al. 2012).

Most known protistan copepod parasites are alveolates (Skovgaard 2014) and euglenozoans (Michajlow 1972); this study suggests that Ascetosporea also harbours a large diversity of copepod parasites and has perhaps been more widely overlooked as parasites of other planktonic crustaceans. Certainly their prevalence and diversity in environmental samples merits further investigation. The morphological similarity of paradinid copepod parasites with those elsewhere in the eukaryote tree of life is a further example of striking levels of convergent evolution in protist (and particularly protistan parasite) evolution. An analogous case is the similarity between the cercozoan and stramenopile diatom parasites, *Pseudopirsonia* and *Pirsonia* respectively. Large‐scale environmental sequencing studies are revealing massive radiations of lineages for which little morphological information is available, but increasingly, parasites are being characterized within these radiations (e.g. Lima‐Mendez et al. 2015), suggesting that much of this newly detected protistan diversity is parasitic. Syndineans and perkinsids are powerful examples of this (Chambouvet et al. 2014, 2015; Guillou et al. 2008), and the diversity revealed in this paper adds to this. We also provide additional evidence that lineage‐specific primers are often able to detect higher levels of diversity and/or lineages that are not amplified by broadly targeted 18S primers, and are an important tool for revealing parasite diversity, activity, and evolution (Bass et al. 2015; Hartikainen et al. 2014a,b; Ward et al. 2016).
